# Unraveling Ruminant Feed Efficiency Through Metabolomics: A Systematic Review

**DOI:** 10.3390/metabo14120675

**Published:** 2024-12-03

**Authors:** Alanne T. Nunes, Camila A. Faleiros, Mirele D. Poleti, Francisco J. Novais, Yamilé López-Hernández, Rupasri Mandal, David S. Wishart, Heidge Fukumasu

**Affiliations:** 1 Department of Veterinary Medicine, School of Animal Science and Food Engineering (FZEA), University of São Paulo, Pirassununga 13635-900, Brazil; alanne.nunes@usp.br (A.T.N.); camilafaleiros@usp.br (C.A.F.); mirelep@usp.br (M.D.P.); 2 Department of Agricultural, Food and Nutritional Science, Faculty of Agricultural, Life and Environmental Science, University of Alberta, Edmonton, AB T6G 2P5, Canada; denovais@ualberta.ca; 3 Departments of Biological Sciences and Computing Science, University of Alberta, Edmonton, AB T6G 2P5, Canada; yamile@ualberta.ca (Y.L.-H.); rmandal@ualberta.ca (R.M.); dwishart@ualberta.ca (D.S.W.); 4 Metabolomics and Proteomics Laboratory, CONAHCyT-Autonomous University of Zacatecas, Zacatecas 98066, Mexico

**Keywords:** livestock production, metabolites, molecular markers, sustainability

## Abstract

Background: Advancements in metabolomic technologies have revolutionized our understanding of feed efficiency (FE) in livestock, offering new pathways to enhance both profitability and sustainability in ruminant production. Methods: This review offers a critical and systematic evaluation of the metabolomics methods used to measure and assess FE in ruminants. We conducted a comprehensive search of PubMed, Web of Science, and Scopus databases, covering publications from 1971 to 2023. This review synthesizes findings from 71 studies that applied metabolomic approaches to uncover the biological mechanisms driving interindividual variations in FE across cattle, sheep, goats, and buffaloes. Results: Most studies focused on cattle and employed targeted metabolomics to identify key biomarkers, including amino acids, fatty acids, and other metabolites linked to critical pathways such as energy metabolism, nitrogen utilization, and muscle development. Despite promising insights, challenges remain, including small sample sizes, methodological inconsistencies, and a lack of validation studies, particularly for non-cattle species. Conclusions: By leveraging state-of-the-art metabolomic methods, this review highlights the potential of metabolomics to provide cost-effective, non-invasive molecular markers for FE evaluation, paving the way for more efficient and sustainable livestock management. Future research should prioritize larger, species-specific studies with standardized methods to validate identified biomarkers and enhance practical applications in livestock production systems.

## 1. Introduction

Increasing livestock efficiency is a farm production strategy aimed at enhancing the availability of livestock products while reducing competition for land, food, and other resources between livestock and humans [1,2]. This strategy also attends to the growing demand for sustainable livestock production and reduction in the environmental footprint of livestock farms and ranches [2,3]. The selection of animals with high feed efficiency (FE), which have a greater capacity to transform low-value, low-protein food resources into high-value, high-protein food products, is expected to increase both food quality and food production, thereby helping to feed the ever-growing human population while decreasing methane emissions and mitigating climate impacts over the long term [4,5,6,7,8]. Additionally, since feed costs account for up to 70% of total livestock production expenses, enhancing FE can significantly reduce production costs, boost profitability, and improve the economic sustainability of livestock farming [9,10].

To accurately estimate feed efficiency (FE), several measures can be used [5], with the most common being feed conversion ratio (FCR) and residual feed intake (RFI). The FCR is defined as the ratio between dry matter intake (DMI) and average daily gain (ADG), where animals with a higher FCR are considered less efficient [11]. The RFI is calculated as the residual from a regression of the DMI on metabolic body weight (MBW = BW0.75) and ADG. Other variables can be included in the regression model, such as ultrasound measurements to estimate fat and protein gain and feeding frequencies [12,13,14]. Other FE traits that may be used with ruminants include the ADG residual, the Kleiber ratio, the relative growth rate, and the feed efficiency index. The ADG residual is calculated as the residual from a regression of the ADG on MBW and DMI, with more efficient animals showing higher residual values [15]. The Kleiber ratio is defined as ADG per unit of MBW, which considers increased ADG without a corresponding increase in MBW, thereby leading to growth without additional maintenance energy costs [16]. The relative growth rate is calculated as the logarithm of MBW at the end of the test period minus the logarithm of MBW at the beginning, divided by test days [17]. The feed efficiency index is calculated as the difference between the recorded and predicted DMI, where the predicted DMI is the mean predicted DMI over the entire experimental period [18]. For dairy animals, energy-corrected milk (ECM) divided by DMI [19] can also be used, where ECM yield is calculated as ECM yield = [0.327 × milk yield (kg/d)] + [12.95 × milk fat yield (kg/d)] + [7.2 × milk protein yield (kg/d)] [20]. Most of these traits require individual measurements of feed intake and animal performance (MBW or milk yield) over a period of 56 to 70 days to achieve accurate estimates [21,22].

Despite being a topic of great interest for more than a century [23], selection for FE remains a challenge because of the complexity of interactions at the molecular, cellular, and tissue levels, and between biological systems [24,25]. The FE is affected by at least five biological processes: (1) feed intake, (2) digestion, (3) metabolism (anabolism and catabolism associated with variations in body composition), (4) physical activity, and (5) thermoregulation [25,26]. The high cost of obtaining FE data and the difficulty in defining well-characterized populations for FE evaluation have not only limited its large-scale application in livestock farming [27] but have also increased interest in exploring the molecular basis of FE through genomics, transcriptomics, proteomics, and metabolomics [25,26]. Using omics techniques to assess FE offers the potential to acquire a comprehensive understanding of the biological mechanisms connected to FE while at the same time allowing the identification of potential low-cost molecular markers for high FE [28,29,30,31,32].

Among the available omics methods for FE evaluation or prediction, metabolomics appears to be particularly promising. Metabolomics is a field of omics science that focuses on the comprehensive, high-throughput characterization of small molecules (i.e., metabolites) in different organisms, tissues, or systems [33]. Metabolites are the downstream products of the molecular-scale activities encoded by the genome and affected by the proteome. Metabolite levels are also heavily influenced by the macro-scale inputs from the environment (diet, weather, stress) in which an organism lives. The fact that the metabolome represents the convergent effects of both the environment and the genotype means that metabolomics is a particularly powerful technique for measuring the molecular phenotype of an organism.

The appeal of metabolomics for evaluating FE in ruminants lies in the fact that it allows the early, routine, and non-invasive identification of phenotypic traits that are otherwise too costly or too time-consuming to measure [34]. In addition, metabolomics offers considerable potential for identifying molecular markers of FE, which has attracted significant interest from livestock researchers [34,35,36,37,38,39]. In particular, the ability to identify low-cost, easy-to-measure blood, fecal, or urinary metabolite biomarkers of FE would be a significant game-changer for the livestock industry [27]. Two different methods are used by metabolomics researchers exploring FE and identifying FE biomarkers: untargeted metabolomics and targeted metabolomics. Untargeted metabolomics assumes no prior knowledge of the metabolome [40,41] and, therefore, offers broad metabolite coverage. However, it is a relatively labor-intensive and time-consuming approach and rarely yields quantitative data [40,42,43,44]. Untargeted metabolomics is often used for biomarker discovery or broad metabolite surveys. It typically uses techniques such as liquid chromatography (LC) coupled with high-resolution mass spectrometry (HRMS) using Quadrupole Time-of-Flight (QTOF) or Orbitrap mass spectrometers. On the other hand, targeted metabolomics focuses on measuring a set of specific, well-defined metabolites [45]. It can be quite fast, is suitable for automation, and can be fully quantitative. Targeted metabolomics is an ideal approach for biomarker validation and implementation [40]. It typically uses techniques such as LC coupled with tandem mass spectrometry (MS/MS), nuclear magnetic resonance (NMR) spectroscopy, and gas chromatography–mass spectrometry (GC-MS). Other methods used in targeted metabolomics can include inductively coupled plasma mass spectrometry (ICP-MS), radioimmunoassay (RIA), enzyme-linked immunosorbent assay (ELISA), fluorometric assay, and colorimetric assay.

The present review is aimed at providing a critical and systematic evaluation of the scientific literature related to the available applications of different metabolomics methods for FE measurement and assessment in ruminants. We discuss the main techniques used, the types of samples analyzed, the metabolites and metabolic pathways identified with FE, along with objective assessments of the study limitations. In doing so, we hope to provide useful insights and thoughtful perspectives on how metabolomics can and should be used for enhancing the molecular understanding of the FE, detecting FE biomarkers, and assisting with FE selection. To the best of our knowledge, this is the first review to evaluate state-of-the-art metabolomic techniques applied to the evaluation of livestock FE. The perspectives presented aim to guide future research on the application of cutting-edge metabolomic technologies to enhance animal productivity, optimize nutritional strategies, and refine precision breeding programs, ultimately paving the way for more efficient and sustainable livestock management.

## 2. Materials and Methods

This systematic review was performed in accordance with the Preferred Reporting Items for Systematic Reviews and Meta-Analyses (PRISMA) guidelines [46]. To compile this systematic review, a combination of web-accessible data mining tools and manual curation to survey peer-reviewed journal articles on the subjects of FE and metabolomics covering the period from 1971 to 2023 was used. All retrieved publications were manually reviewed using Start (State of the Art through Systematic Review) [47] at the Universidade Federal de São Carlos (São Carlos, SP, Brazil).

### 2.1. Search Strategy

We used PubMed (https://pubmed.ncbi.nlm.nih.gov/ (accessed on 16 November 2023)), Web of Science (https://www.webofscience.com/wos/woscc/basic-search (accessed on 16 November 2023)), and Scopus (https://www.scopus.com/search/form.uri?display=basic#basic (accessed on 16 November 2023)) databases to identify metabolomic studies relevant to FE in ruminants. Keywords were categorized into three groups: metabolomic studies (metabolomics, metabonomic, metabolome, or metabolite), ruminant species (ruminant, cattle, beef cattle, dairy cattle, bull, heifer, steer, calf, sheep, goat, lamb, or buffalo), and FE-related phenotypes (feed efficiency, feed conversion, feed conversion rate, residual average daily gain, or residual feed intake). Boolean operators were applied to combine these terms.

### 2.2. Selection Criteria and Data Extraction

The initial search generated a broad list of results, which were screened by title and abstract to exclude duplicates, reviews, and studies unrelated to FE or ruminants. Studies reporting the association between metabolites and FE measurements in ruminants were selected for full review. Only English-language studies were included. Key data were extracted, including metabolomics approaches, analytical platforms, sample types, animal characteristics, FE parameters, metabolites identified, and references.

### 2.3. Paper Screening Process

**Figure 1:**
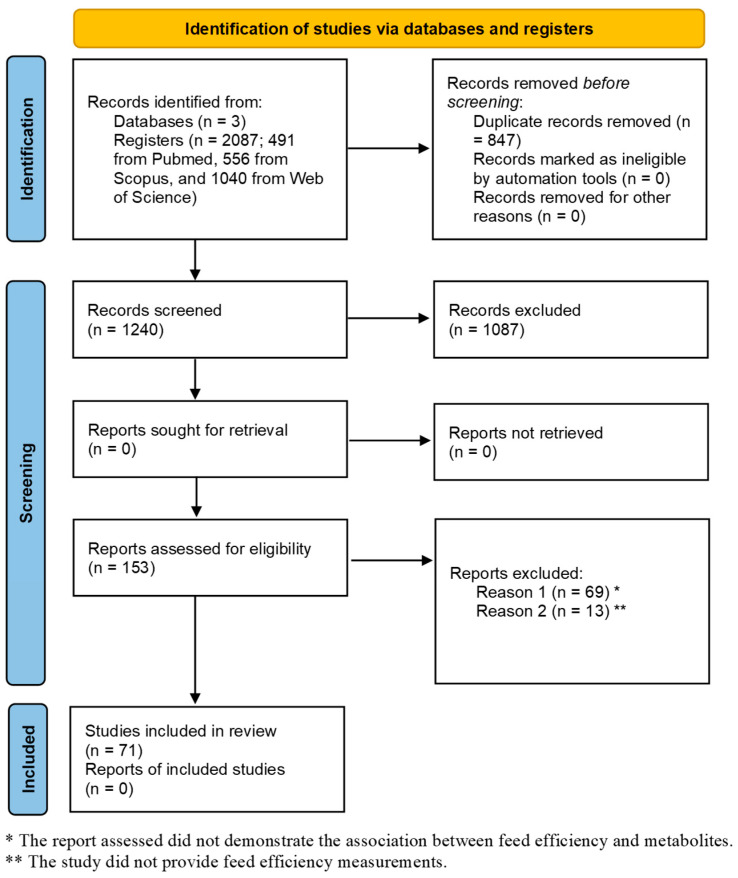
PRISMA diagram. The preferred reporting items for systematic reviews and meta-analysis (PRISMA) flow diagram identified the total number of articles initially surveyed and the number of articles included and excluded from this systematic review.

From the initial set of 2087 articles found in the three literature databases, 153 remained potentially eligible for inclusion in our study after applying the exclusion criteria. Abstract screening reduced the target number of peer-reviewed manuscripts to 84. The selected publications were either freely available as open access or obtained through institutional subscriptions. All the papers were carefully read, and the final number was further reduced to 71 by selecting only those papers that reported associations between FE and metabolite measurements as well as those papers that included FE trait evaluation. Detailed information on keyword selection, search engines, databases, journals, and search strategies is provided below and summarized in the Preferred Reporting Items for Systematic Reviews and Meta-analysis (PRISMA) [46] checklist and flowchart (Figure 1). Details of the titles, authors, and years of publication of the selected studies are presented in Supplementary Table S1.

### 2.4. Association Between Metabolites and Feed Efficiency

Metabolites associated with FE parameters were analyzed with respect to metabolic pathways to enable a more detailed discussion of the results or findings obtained from the selected studies. Metabolic pathway analysis was carried out using MetaboAnalyst software (version 6.0) [48], an online processing tool for metabolomic data, to identify the predominant pathways associated with FE.

Simple correspondence analysis was applied to investigate the potential associations between the reported analysis platform and the metabolites identified as associated with FE. To ensure robustness and relevance, only metabolites that appeared two or more times in the literature were included in this analysis. The data were organized into a contingency table, and the analysis was conducted in Python using the ‘prince’ library. A chi-squared test was performed to assess the existence of a statistically significant association between the two categorical variables, after which a correspondence analysis was applied.

## 3. Results

### 3.1. Study Design, Species, and Analytical Approaches in Metabolomics Research

Our systematic analysis of the 71 selected papers revealed significant trends in the geographical distribution, study design, and analytical approaches utilized in metabolomic research on FE in ruminants. The majority of studies (80.3%) were conducted in the United States, Brazil, Canada, and China, reflecting a concentration of research efforts in regions with robust ruminant production industries and advanced research infrastructure. This geographical distribution highlights the prominence of these countries in advancing metabolomic studies while also revealing potential gaps in research activity from other regions. The concentration of studies in these regions suggests a need for global collaboration and the expansion of metabolomic research to underrepresented areas, which could provide a more comprehensive understanding of FE across diverse ruminant production systems.

**Figure 2:**
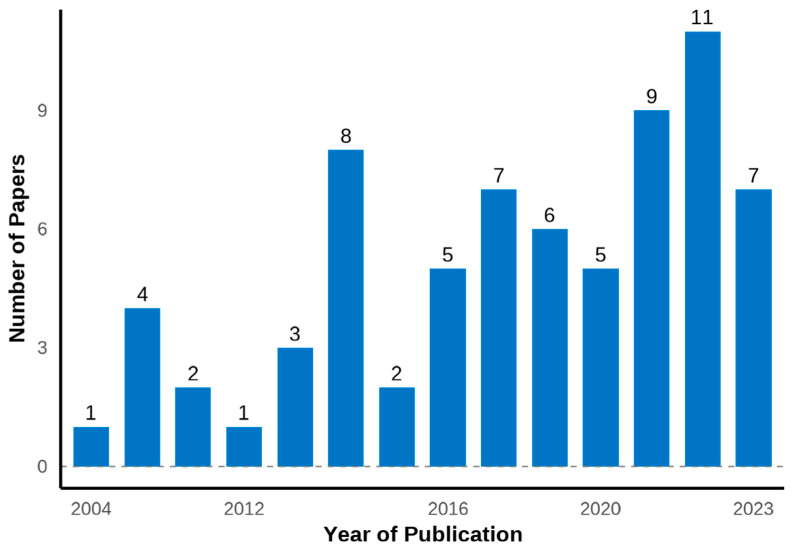
Number of papers published over the years applying metabolomics to evaluate feed efficiency in ruminants.

The analysis of publication trends reveals a notable increase in studies on metabolomic approaches to FE in ruminants over the past decade. Of the 71 selected papers, the majority (84.5%) were published between 2014 and 2023 (Figure 2), indicating a growing interest in this field. This is particularly evident in recent years, with the highest number of publications occurring in 2022 and 2023, collectively accounting for 22% of all studies. Prior to 2014, publications were less frequent and sporadic, with only 11 papers published before this period, including the earliest study in 2004. These trends reflect the increasing recognition of metabolomics as a valuable tool for understanding and improving FE in the livestock sector.

**Figure 3:**
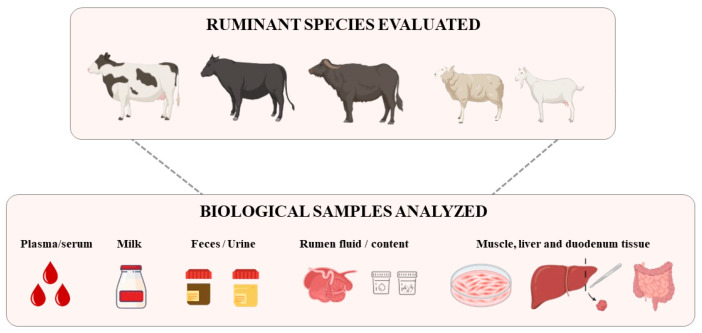
Species and biological samples were evaluated using metabolomic approaches for feed efficiency research in ruminants.

**Figure 4:**
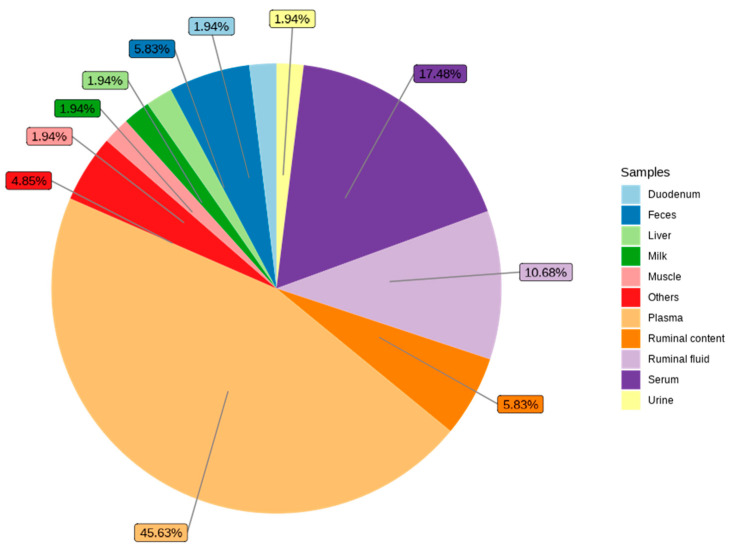
Biofluids or tissues evaluated using metabolomic approaches for feed efficiency research in ruminants. Others included total blood, colostrum, ileum, jejunum, and adipose tissues.

Figure 3 shows the different biofluids and tissues used for metabolomic analysis carried out to understand FE in cattle, sheep, goats, and buffalo. Cattle-based studies (87.3%) significantly surpassed those analyzing sheep (9.9%), goats (1.4%), and buffaloes (1.4%). A considerably greater number of studies on beef cattle were observed relative to dairy cattle, with the former accounting for 79% of cattle-based studies. Regarding biological samples used for metabolomic studies, plasma was the most commonly analyzed biofluid (45.6%), followed by serum (17.5%) and ruminal fluid (10.7%) (Figure 4). The prominence of plasma, in particular, underscores its utility in providing a systemic overview of metabolic processes, making it a valuable resource for identifying biomarkers associated with FE.

Targeted metabolomics methods were applied in approximately 59% of the selected studies, whereas the untargeted approaches were applied to 34%. The remaining 7% employed both approaches. Regarding the technology used for metabolite analysis, liquid chromatography–mass spectrometry (LC-MS) was utilized in 26.8% (19/71) of the studies. Of these, studies employing HRMS with QTOF or Orbitrap instruments dominated LC-MS-based analyses (84.2%, 16/19 studies), while 15.8% (3/19 studies) applied MS/MS methods with triple quadrupole or Qtrap instruments, reflecting advancements in metabolomic technologies.

NMR was employed in 11.3% (8/71 studies) and GC-MS in 7% (5/71 studies). Other techniques, such as electrospray ionization mass spectrometry (ESI-MS/MS), ICP-MS, and direct-injection mass spectrometry (DI-MS), were used in 8.4% of the studies. Additionally, 18.3% of the studies applied chromatographic methods, including gas and high-performance liquid chromatography coupled with other detection methods. The remaining 28.2% used specialized automated chemical analyzers based on fluorometric and colorimetric assays, RIA, and ELISA to measure specific compound concentrations. Notably, 46.4% of studies combined multiple analytical methods, indicating a general reliance on fragmented approaches rather than comprehensive methodologies capable of capturing a holistic metabolic profile.

### 3.2. Association Between Metabolites and Feed Efficiency

To estimate FE, RFI was the primary trait evaluated in the reviewed studies, accounting for 71.8% of the selected publications. Its widespread adoption reflects its effectiveness as an FE measure, as it accounts for variations in maintenance and production energy requirements, making it a reliable indicator of true feed efficiency. In contrast, the FCR, a traditional metric influenced by factors such as body weight and production levels, was used in only 7% of the studies. Alternative FE measurements, which collectively appeared in 22.3% of the studies, included traits such as the feed efficiency index, average daily gain (ADG) within a specific dry matter intake (DMI) range, ADG residual, Kleber Ratio, energy-corrected milk (ECM) divided by DMI, and relative growth rate. This diversity of FE traits highlights the complexity of evaluating feed efficiency across different production systems and species. However, the lack of standardization in these alternative measures complicates cross-study comparisons and underscores the need for more unified and consistent FE assessment methodologies.

**Figure 5:**
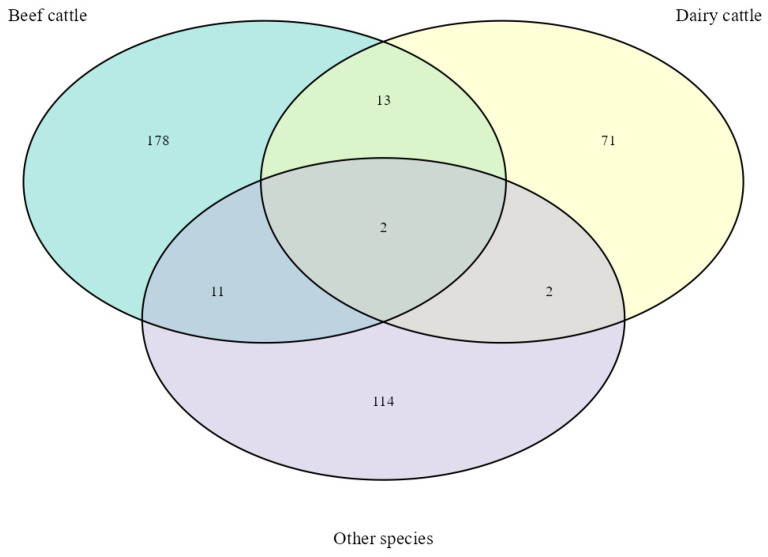
Total of metabolites related to feed efficiency in ruminants. Venn diagrams representing the core unique and shared metabolites between species/categories evaluated. “Other species” included sheep, goats, and buffaloes.

Figure 5 illustrates the metabolites shared among the species/categories evaluated in the studies selected for this review. The “Other species” category includes studies involving sheep, goats, and buffaloes. Overall, 391 metabolites were associated with FE across all species and categories. Among this set of 391, only 13 (3.3%) metabolites were detected in both beef and dairy cattle, 11 (2.8%) metabolites were identified in beef cattle and other species, and two (0.5%) metabolites were found in dairy cattle and other species. Only two metabolites (0.5%) were common across all evaluated species.

Table 1 summarizes the information available on the selected studies, including the species, animal category (dairy, beef, etc.), sample size, FE traits, and relevant findings of the selected studies. The average number of animals measured in the selected papers was 83.9 ± 105.60 animals, with the smallest number being 10 and the largest number being 493.

**Figure 6:**
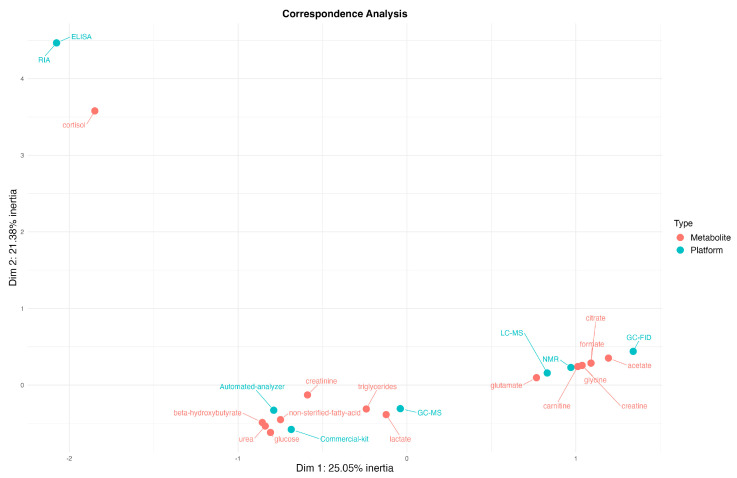
Simple correspondence analysis between the analysis platform and metabolites associated with feed efficiency in ruminants.

Regarding the investigation of the potential associations between the reported analysis platform and the metabolites identified as associated with FE, we conducted a chi-square test, which revealed a significant association (*p* < 0.05) between the type of platform used and the metabolites reported. Following this, we performed a correspondence analysis (Figure 6) to further explore these associations. This analysis revealed that 25.05% of the variance is explained by the first dimension on the *X*-axis (Dim 1) and 21.38% by the second dimension on the *Y*-axis (Dim 2), totaling 46.43% of the variance explained. The principal component on the *X*-axis represents the separation of metabolomics platforms based on metabolite detection patterns, while the *Y*-axis highlights secondary variances, potentially grouping platforms and metabolites based on more specific detection biases or metabolite categories. We identified three main clusters in the analysis. The RIA and ELISA methods were strongly associated with cortisol, forming a distinct group. The LC-MS and NMR methods clustered together, predominantly grouping amino acids. The automatic analyzer and commercial kits were closely linked to fatty acids, carbohydrates, and ketone bodies. Additionally, gas chromatography methods (GC-MS and GC-FID, gas chromatography coupled with flame ionization detector) were predominantly associated with lactate, fatty acids, and rumen short-chain fatty acids. These results demonstrated that the choice of analytical platform can bias the identification of potential biomarkers for FE.

#### Key Biological Pathways Linked to Feed Efficiency Biomarkers

**Figure 7:**
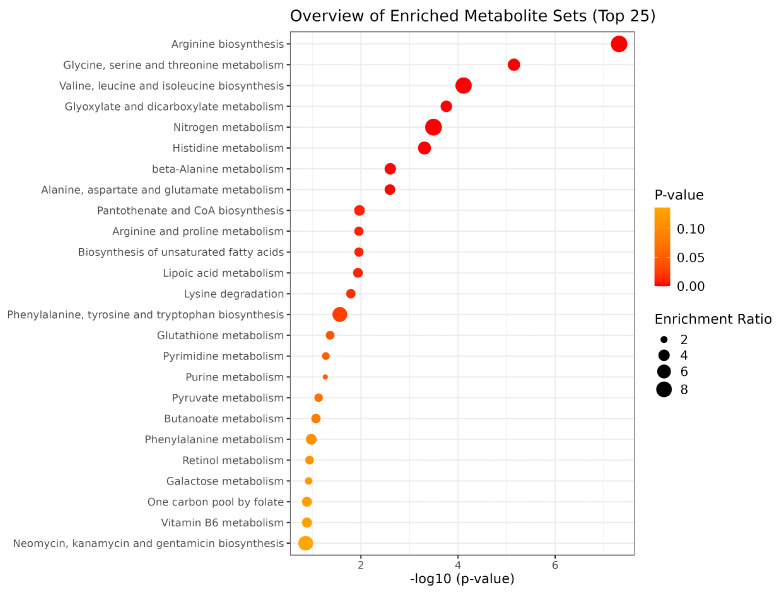
Overview of enriched metabolite sets (top 25) associated with feed efficiency in ruminants. The graph was generated using MetaboAnalyst 6.0.

The results of enrichment analysis demonstrated that amino acid pathways, including pathways associated with arginine, glycine, serine, threonine, valine, leucine, isoleucine, histidine, beta-alanine, alanine, aspartate, glutamate, and proline, were significantly (*p* < 0.05) associated with FE. Additionally, the pathways associated with pantothenate, nitrogen metabolism, and fatty acid biosynthesis were also significantly (*p* < 0.05) associated with FE (Figure 7).

The association between specific metabolites and FE was analyzed, revealing their involvement in key biological pathways. Metabolites such as glucose, amino acids, and fatty acids were consistently reported across multiple studies, highlighting their importance in FE. For instance, branched-chain amino acids and lipids were frequently linked to muscle development and fat deposition, while urea and nitrogen-related metabolites were strongly associated with protein turnover. This systematic mapping underscores the complex interplay of metabolic pathways contributing to variations in FE in ruminants.

## 4. Discussion

### 4.1. Study Design, Species, and Analytical Approaches in Metabolomics Research

The selection of feed efficiency (FE) in ruminants has been the focus of extensive research over the past century [23]. However, owing to the complexity of FE traits and the high costs associated with obtaining accurate data and defining well-characterized populations [27], achieving significant advances in FE selection continues to be challenging. These challenges have driven efforts to apply metabolomics analyses to deepen our understanding of the molecular basis underlying individual variations in FE [25,26]. Metabolomics has the potential to identify FE molecular markers and to elucidate metabolic pathways related to FE [112,113]. In this review, we examined 71 studies conducted in 15 countries, with a particular emphasis on metabolomics as a tool for understanding FE in ruminants. Our primary objectives were to identify the metabolites associated with FE with potential use as biomarkers and to provide insights and perspectives on the application of metabolomics for FE selection.

The metabolomics studies included in this review were typically performed in cattle (87.3%), significantly surpassing studies in sheep, goats, and buffaloes. The same was observed in a previous review, in which cattle-based studies surpassed all other species evaluated [114]. Goldansaz et al. (2017) demonstrated that metabolomics can be applied to cattle to investigate potential molecular biomarkers related to animal diseases, reproductive performance, and the assessment of production traits, which can be useful as a gold standard for understanding traits of interest in other livestock species. However, our results also indicated that only a small number of metabolites related to FE are shared across different ruminant species. Given the genetic diversity within livestock populations and the distinct environmental adaptations of various ruminant species, it is essential to conduct species-specific studies to gain a deeper understanding of FE in ruminants [115,116]. Several metabolomics databases developed by The Metabolomics Innovation Centre (TMIC) at the University of Alberta are already accessible to ruminants. These include the Livestock Metabolome Database (LMDB) [115], the Milk Composition Database (MCDB) [117], and the Bovine Rumen Metabolome Database (RMDB) [118]. These databases were developed to aid in livestock metabolomics research efforts.

Regarding the biological samples evaluated, our results showed that most FE metabolomics studies used well-studied biofluids such as serum, plasma, urine, milk, and rumen content or liquid [44,114]. The most frequently evaluated biofluids were plasma (45.6%) and serum (17.4%). Plasma has previously been reported as the most widely examined sample in metabolomics research, possibly reflecting the ease of sampling but also its potential usefulness as a surrogate reporter for all organs of the body [119]. Blood is the main route of absorption and transport of nutrients to organs and tissues, with the transported metabolites being involved in various metabolite processes, which increases the potential for biomarker discovery [70].

Considering the metabolomic approach applied in the selected studies, despite a significant increase in untargeted metabolomic studies during the past decade [120], our results showed a greater number of targeted metabolomic studies applied to FE in ruminants. Among the analytical tools used for targeted approaches, mass spectrometry (MS) analyses are particularly prominent [121]. Various MS methods are available [122], including LC-MS, which involves scanning samples sequentially with a mass spectrometer during chromatography. This process generates a time series of spectra, each containing a list of ions with a mass-to-charge ratio (*m*/*z*) and intensity values [123]. LC-MS analyses were performed in more than 20% of the selected studies in this review.

Furthermore, 28.2% of the selected studies applied specialized automated analyzers based on fluorometric and colorimetric assays, as well as RIA and ELISA, to measure specific compound concentrations. The preference for these technologies may be due to the fact that these methods are simpler, cheaper, and can be performed in non-specialized laboratories [124]. However, these techniques typically measure only a handful of compounds and have a narrow dynamic range, limiting their ability to accurately quantify molecules at very high or very low concentrations [124,125,126,127]. As indicated by the correspondence analysis, the molecules associated with FE measured using these platforms showed no association with those identified by metabolomics-specific methods.

### 4.2. Association Between Metabolites and Feed Efficiency

To estimate FE in ruminants, our results showed a considerable variation in the choice of traits, as previously noted by Berry and Crowley (2013). The most popular FE traits are residual RFI and FCR. The FCR is defined as the DMI divided by the ADG; animals with a lower FCR are reported to be more efficient [128]. Although FCR has been the most commonly used FE measure in the last decades [5], FCR selection may not be optimal. In particular, FCR is dependent on ruminant production characteristics, meaning that it is sensitive to the level of production related to net FE, which is associated with an increase in the mature size of female progeny. This dependency can cause an undesirable increase in maintenance energy costs [129]. The RFI, also known as net feed efficiency [130], involves partitioning feed inputs into maintenance and production components, allowing the quantification of inter-animal variation in feed intake compared to the feed required for maintenance and production [131]. As RFI shows moderate heritability [29] and is phenotypically independent of the production characteristics used for its estimation, it can be used to compare animals at different production levels [26,132], which has led to a growing preference for RFI over FCR [27].

**Figure 8:**
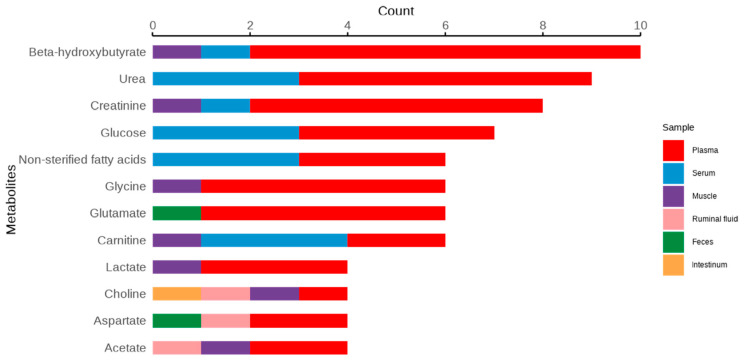
TOP 12 metabolites associated with feed efficiency in ruminants in the studies selected in this review and the biological samples in which they were detected.

The metabolites that were mostly related to FE in the selected studies, as well as the biological samples in which they were evaluated, are shown in Figure 8. The 12 common metabolites mostly reported to be associated with FE included beta-hydroxybutyrate (BHBA), urea, creatinine, glucose, carnitine, glutamate, glycine, non-esterified fatty acids (NEFA), acetate, aspartate, choline, and lactate.

Our enrichment analysis results, conducted using all 391 significantly altered metabolites reported by the 71 studies, also highlighted the importance of a number of amino acid (AA) metabolic pathways, including those involved in arginine, glycine, serine, threonine, valine, leucine, isoleucine, histidine, beta-alanine, alanine, aspartate, glutamate, pantothenate, and proline metabolism. This analysis also identified the importance of metabolic pathways associated with nitrogen metabolism and fatty acid biosynthesis. These results agree with previous studies that indicated nitrogen metabolism as a crucial key factor for differential FE in ruminants [103,133,134]. In ruminants, nitrogen metabolism is particularly complex. Dietary proteins are broken down into peptides and AAs, leading to the formation of ammonia, which contributes to nitrogen loss. Therefore, the efficiency of nitrogen retention directly affects overall nitrogen utilization [135]. Additionally, because AAs undergo microbial degradation in the rumen, their metabolic pathways become somewhat more complex, reducing the accuracy of predictions regarding the quality and quantity of absorbed AAs [136]. This metabolic complexity also explains the significant interactions between AA metabolism, the microbiome, and nutrient utilization in livestock and their corresponding association with FE [39,137,138].

Differences in microbial populations have been shown to influence AA absorption, as some microbes secrete metabolites that interact with gastrointestinal receptors, thereby regulating nitrogen utilization efficiency in the host [139]. Changes in the microbiome can optimize AA absorption in feed-efficient animals, altering the levels of AAs that play a role in energy metabolism through gluconeogenesis. These AAs include glycine, glutamate, phenylalanine, threonine, tyrosine, and proline [77]. On the other hand, variations in branched-chain amino acid (BCAA) profiles, such as the concentrations of valine, leucine, and isoleucine, can affect the synthesis of rumen microbial protein, which accounts for up to 90% of the amino acids reaching the small intestine [140].

In addition, FE research examining the link between the metabolome and microbiome has shown that inefficient animals have inefficient microbiomes, which are characterized by a greater diversity of species, genes, and KEGG pathways. This diversity leads to a wider range of metabolites being generated. In contrast, efficient animals typically exhibit a simpler microbiome with more streamlined metabolic pathway networks and an increased dominance of a smaller number of functional metabolic components [103,133,134]. Consequently, the microbiome and metabolome of efficient animals are less complex but more specialized in fulfilling the host’s energy requirements [103]. It has been reported that the rumen microbiome of inefficient ruminants exhibits higher levels of nitrogen metabolism, as evidenced by more active pathways related to cysteine, histidine, lysine, methionine, and tyrosine [103,134].

As building blocks of proteins, AAs are essential for many key physiological functions, including growth, reproduction, lactation, and maintenance [136]. Given the role of specific AAs as biomarkers of FE in ruminants, the studies reviewed here indicate that their effect on this phenotype is largely due to their involvement in metabolic pathways related to protein synthesis, muscle growth, and overall energy efficiency. Although some differences have been observed between FE studies, it has been noted that efficient animals show greater muscle mass, associated with higher plasma creatinine levels [52,57], and a lower rate of protein degradation, represented by lower plasma urea [55,61]. In addition, in more efficient animals, certain urea cycle metabolites, such as valine and lysine, are generally reduced [36,141]. These amino acids undergo the initial steps of reversible transamination and irreversible oxidative decarboxylation, entering the urea cycle via transamination to glutamate [142]. The reduction in ureagenesis may suggest improved nitrogen efficiency in more feed-efficient animals [143,144,145,146]. These results potentially indicate that characteristics such as body composition, muscle protein degradation, and N-use efficiency are likely to be common determinants of variations in FE [52,57].

In addition to these findings, it has been consistently reported that circulating concentrations of BCAAs, such as isoleucine and valine, are higher in high RFI animals [36,57,92]. These metabolites can impair insulin action and enhance adiposity in less efficient animals by increasing lipid synthesis and fat accretion [92], as they facilitate the passage of fatty acids across mitochondrial membranes to be degraded by oxidation. The BCAAs also mediate the activation of hepatic metabolic pathways [147]. Conversely, because BCAAs in the rumen are indicative of increased microbial protein in the rumen, which is used for milk protein synthesis in the mammary gland, the enrichment of BCAA degradation pathways has been previously linked to lower efficiency in dairy cows [133].

Regarding energy metabolism, ruminants are distinct because gluconeogenesis serves as the main source of glucose, unlike non-ruminants, which primarily absorb glucose directly from their diet [148,149]. Within this metabolic framework, carnitine and glucose have been identified as key metabolites associated with FE. Carnitine is an AA that plays an important role in cellular energy metabolism by transferring acyl groups from the cytoplasm to the mitochondrial matrix for β-oxidation [150,151]. Carnitine also transports long- and medium-chain fatty acids (LCFAs and MCFAs) into the mitochondria through interactions with fatty acid-bound coenzyme A [152]. In addition, carnitine regulates the metabolism of lipids and glucose, rendering the utilization of energy in feed and body stores more efficient [153], which may influence body composition [154]. Carnitine is synthesized endogenously, but several precursors and cofactors are required, including lysine, methionine, vitamin C, niacin, vitamin B12, choline, and reduced iron [152]. Although carnitine supplementation has been previously shown to increase glucose concentrations [155], suggesting improved nutrient utilization, the selected studies in this review reported either no association [87] or slight positive associations [77] between carnitine concentrations and RFI in cattle.

Findings on glucose concentrations and their relationship with FE are inconsistent. While some studies have reported a negative correlation between glucose levels and feedlot RFI [72,95], research on calves [75], growing animals [81], and pregnant cows [55] found no association. Additional research has shown that glucose concentrations are negatively related to both RFI [36,54] and feed gain ratio [78]. The observed discrepancies may be attributed to variations in sample handling. For example, glucose levels can fluctuate significantly depending on the duration samples are left at room temperature before freezing, as glycolytic reactions in serum or plasma can quickly convert glucose to lactate [36]. In addition, blood glucose concentrations are considered insensitive markers of energy status in cattle because of their homeostatic regulation [156,157,158]. That is, glucose concentrations are usually maintained within a physiological range (40–60 mg/dL in cattle [157,159], 25–50 mg/dL in sheep [160], 65–88 mg/dL in goats [161], and 25–90 mg/dL in buffaloes [162,163]) by the mobilization of carbohydrates through glycogenolysis, lipolysis, and gluconeogenesis [164].

Regarding the association between fatty acids and FE in ruminants, it is possible to highlight BHBA and NEFA as potential molecular markers. These metabolites have been reported as useful biochemical indicators of energy metabolism and nutritional status [165] because they arise from the metabolism of adipose tissue, particularly during periods of fasting or high-energy requirements, such as lactation [166,167]. Ketone bodies, such as BHBA, are oxidized in the muscle [168] and used as energy substrates by muscles in ruminants, with extraction rates by hindlimb muscles ranging from 10 to 45% (superior to glucose) [169]. As BHBA is a byproduct of tissue-derived fatty acid catabolism, its systemic concentrations increase proportionally with the degree of fat mobilization [61] and are inversely related to subcutaneous lumbar and rump fat accretion [81]. Moreover, BHBA is the most stable ketone body and is commonly used as a biomarker of energy balance [170]. Our results demonstrated that plasma concentrations of BHBA were negatively associated with RFI in lactating cows, suggesting that the nutrient and energy intake of efficient animals was sufficient to meet nutritional needs during lactation [79] but positively associated with RFI and DMI in growing animals [61,72].

Finally, the relationship between NEFA concentration and FE yielded mixed results. In growing animals, NEFA concentrations either showed no association with RFI [67,81,84,92] or were negatively associated with RFI [61], with no clear explanations provided. Similarly, no association was found between NEFA concentrations and RFI in bulls [78]. However, a negative association was observed in pregnant beef cows [55] and heifers [171], suggesting that differences in fat mobilization may be required to support oxidative metabolism in maternal tissues. This indicates that the ability of pregnant cows to mobilize fat may play an important role in the FE of pregnant animals [55].

#### Key Biological Pathways Linked to Feed Efficiency Biomarkers

**Figure 9:**
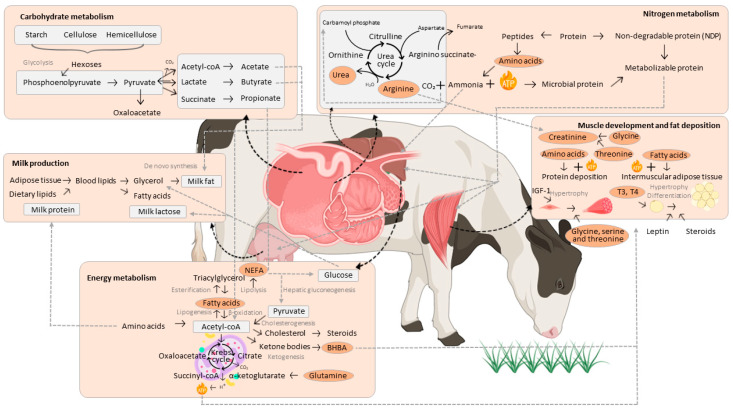
Metabolites associated with the main biological processes in ruminants. Molecules associated with feed efficiency in ruminants in the studies selected for this review are highlighted in orange ellipses.

We summarized the metabolites associated with FE identified in the reviewed studies, grouped by their respective roles in carbohydrate metabolism, nitrogen metabolism, energy metabolism, milk production, muscle development, and fat deposition (Figure 9). Carbohydrate metabolism includes especially short-chain fatty acids, which play essential roles in energy production and utilization in ruminants [172]. Nitrogen metabolism involves amino acids and urea, essential for protein turnover and the recycling of nitrogen [173]. Energy metabolism is driven by compounds such as fatty acids, NEFA, and BHBA, which fuel processes like lipogenesis, ketogenesis, and cholesterol synthesis [167]. These metabolites ensure that energy demands are met for growth and production.

Milk production involves compounds such as glycerol, which contributes to milk fat synthesis, and amino acids, which support the production of milk protein and lactose [174]. Muscle development and fat deposition rely heavily on amino acids, such as glycine and BCAAs, as well as fatty acids, to support protein deposition and the formation of adipose tissue [92,175]. Hormonal mediators, including IGF-1 and thyroid hormones, also play pivotal roles in regulating these processes [176]. Collectively, these pathways demonstrate the metabolic complexity underlying FE, providing insights into how metabolomic studies have identified specific molecules as potential biomarkers for selecting FE in ruminants.

### 4.3. Limitations and Strength

The application of metabolomic technologies presents promising opportunities for evaluating and selecting ruminants with superior FE, offering a cost-effective alternative to traditional manual measurements [24]. Over the past decade, more than 70 studies have utilized metabolomic approaches to investigate the biological mechanisms underlying variations in FE among ruminant species. These studies demonstrate the potential of metabolomics to identify biomarkers, such as specific amino acids and fatty acids, which play key roles in metabolic pathways associated with FE. By identifying these biomarkers, metabolomics offers non-invasive tools to select high-efficiency animals early in life and to guide precision nutrition programs, enabling breeders to customize feed compositions for optimal performance [114,177]. These advancements collectively empower breeders to improve system productivity, enhance sustainability, and achieve better economic outcomes through data-driven strategies. While these advancements highlight the strengths of metabolomics in FE research, several limitations must also be addressed.

One major limitation is the limited accessibility of metabolomic analyses, which may hinder their widespread adoption in the livestock industry [178]. Additionally, while targeted metabolomics has demonstrated promising results, the application of non-specific or narrow-scope technologies (e.g., RIA, ELISA, GC-FID) has not been particularly effective in identifying reliable FE biomarkers. The diversity of analytical approaches and a lack of standardization further complicate the identification and validation of robust biomarkers.

A noticeable trend in the reviewed cattle-based FE studies was the overabundance of small-scale, preliminary studies with experimental designs that often lacked statistical robustness [113]. Many findings were not validated, reducing their reliability. The situation is even more pronounced for other ruminant species, such as sheep, goats, and buffaloes, where studies are scarce, limiting the transferability and broader interpretation of findings. While validation studies have become routine in human metabolomics [179,180,181], there is a striking lack of independent cross-validation studies in ruminant FE research. Only one such study has been reported for cattle [34], with none for other ruminant species. These gaps highlight the need for larger, multicenter, and more robust studies that include validation of previously identified biomarkers.

Despite these challenges, metabolomics remains a valuable tool with significant potential to advance FE research. To fully realize its benefits, future studies should focus on analyzing larger, species-specific cohorts of ruminant animals using more comprehensive, fully quantitative metabolomic assays. Adopting this approach will deepen our understanding of the molecular mechanisms underlying FE and ultimately enhance livestock selection and management practices.

## 5. Conclusions

Metabolomic technologies offer transformative opportunities for improving FE in ruminant livestock by enabling the identification of molecular biomarkers. This review highlights significant progress in the field, particularly in the identification of amino acids and fatty acids with potential as FE biomarkers. However, the research landscape reveals clear limitations that must be addressed. To fully realize the potential of metabolomics in livestock production, future studies should prioritize standardized, species-specific, and large-scale approaches. These efforts will provide more robust biomarkers, deepen our understanding of FE’s molecular underpinnings, and ultimately enhance livestock selection and management practices.

## Figures and Tables

**Table 1:** Species, category, sample size, feed efficiency traits, and metabolites associated to feed efficiency in ruminants.

Specie	Category	N *	Trait	Metabolites Associated with Feed Efficiency	Reference
Cattle	Beef cattle	112	RFI	Acetate, betaine, carnitine, citrate, creatinine, formate, glycine, glutamate, hippurate, hydroxybutyrate, phenyalanine, threonine, tyrosine	Karisa, Moore and Plastow (2014) [34]
	Beef cattle	91	RFI	Fecal metabolites of cortisol	Montanholi et al. (2010) [49]
	Beef cattle	245	ADG and G: F	Lactate and total ghrelin	Foote et al. (2014) [50]
	Beef cattle	32	RFI	Cholesterol	Bourgon et al. (2017) [51]
	Beef cattle	46	RFI	Creatinine and urea	Santana et al. (2013) [52]
	Beef cattle	14	FE index	No significant association	Fornazari Neto et al. (2021) [53]
	Beef cattle	17	RFI	Glucose	Clemmons et al. (2023) [54]
	Beef cattle	321	RFI	Urea and NEFA ^1^	Wood et al. (2014) [55]
	Beef cattle	16	RFI	2-Hydroxy-4-methylbenzaldehyde, 4-Chloro-L-lysine, 4-Guanidinobutanal, 5-Aminopentanoic acid, Aminohexanedioic acid, Arginyl-cysteine, Asparaginyl-alanine, Azetidinecarboxylic acid, Benzyl salicylic acid, Butylparaben, Carnosine, Citrulline, Creatine, Creatinine, Cystathionine sulfoxide, Cysteinylglycine disulfide, Glutamate, Glutaminyl-glutamic acid, Glutaminyl-methionine, Glutamyl-glutamine, Histamine, Hydroxylysine, Hydroxyprolyl-cysteine, Imidazoleacetic acid, Isoleucyl-alanine, Isomer 1 of 4-Chloro-L-lysine, Isomer 1 of 5-aminopentanoic acid, Isomer 2 of 4-chloro-L-lysine, Isomer 2 of 5-aminopentanoic acid, Isomer of methionine, Isomer of N-formimino-L-glutamic acid, L-alpha-aspartyl-L-hydroxyproline, Mesalazine, Methionine, Methionyl-glutamic acid, Methylguanidine, Ornithine, Prolyl-methionine, Salsoline-1-carboxylic acid, Taurine, Threoninyl-hydroxyproline and Valyl-glutamate	Taiwo et al. (2022) [56]
	Beef cattle	48	RFI	BCFA ^3^, urea and triglycerides	Jorge-Smeding et al. (2021) [57]
	Beef cattle	32	RFI	Beta-hydroxybutyrate	Guarnido-Lopez et al. (2023) [58]
	Beef cattle	58	RFI	Isovalerate	Hernandez-Sanabria et al. (2010) [59]
	Beef cattle	61	RFI	Cortisol	Bonilha et al. (2017) [60]
	Beef cattle	90	RFI	Beta-hydroxybutyrate and NEFA ^1^	Kelly et al. (2010a) [61]
	Beef cattle	38	RFI	No significant association	Lombardi et al. (2022) [62]
	Beef cattle	54	RFI	δ15N in glycine, aspartate, carnosine, flavin adenine dinucleotide, glycine, sarcosine and serine	Meale et al. (2017) [63]
	Beef cattle	53	RFI	Cholesterol	Broleze et al. (2020) [64]
	Beef cattle	18	RFI	Creatinine, plasmatic urea nitrogen and triglycerides	Singh et al. (2019) [65]
	Beef cattle	73	RFI	Acetate, ammonia, beta-hydroxybutyrate, NEFA ^1^ and propionate	Lawrence et al. (2011) [66]
	Beef cattle	85	RFI	Creatinine	Lawrence et al. (2012) [67]
	Beef cattle	59	RFI	No significant association	Trevizan et al. (2021) [68]
	Beef cattle	16	RFI	2,3 dihydroflavone, limonoate, phytanic acid, retinal, stearic acid, and vomifoliol	Novais et al. (2019) [69]
	Beef cattle	493	RFI	Choline, citric acid, dimethylsufone, glutamine, glycine, isoleucine, lactate, leucine, lysine, tyrosine, and valine	Li et al. (2021) [70]
	Beef cattle	39	RFI	Lactate	Lawrence et al. (2013) [71]
	Beef cattle	38	RFI and FCR	Beta-hydroxybutyrate, bilirubin, creatinine, cortisol, glucose, and urea	Richardson et al. (2004) [72]
	Beef cattle	16	ADG	Cholesterol, LPC-18:1 and PC-18:0/20:3	Artegoitia et al. (2019) [73]
	Beef cattle	65	RFI	2-hydroxybutyrate, 3-hydroxybutyrate, acetate, anserine, carnitine, carnosine, choline, creatine, creatinine, glycine, glycerol, isoleucine, lactate, malonate and NAPDH	Consolo et al. (2021) [37]
	Beef cattle	75	ADG, FCR, and RFI	11-hydroxyprogesterone-glucuronide deoxycholic acid glycine conjugated, androsterone sulfate, dexocholic acid glycine conjugated, progesterone and taurochenodeoxycholate	Artegoitia et al. (2022) [74]
	Beef cattle	18	RFI	Plasmatic urea nitrogen and urea	Sharma et al. (2014) [75]
	Beef cattle	112	RFI	Fecal metabolites of cortisol	Montanholi et al. (2013) [76]
	Beef cattle	112	RFI	Acetate, betaine, carnitine, citrate, creatine, creatinine, formate, glutamate, glycine, hippurate, hydroxyisobutyrate, lysine, phenylalanine, threonine and tyrosine	Karisa et al. (2014) [77]
	Beef cattle	302	RFI, G: F, KR, RGR	Glucose	Kelly et al. (2011) [78]
	Beef cattle	27	RFI	Glucose, Phosphorus (P) and triglycerides	Souza et al. (2019) [79]
	Beef cattle	236	RFI	Corticosterone and cortisol	Foote et al. (2016) [80]
	Beef cattle	50	RFI	Beta-hydroxybutyrate, glucose, and urea	Kelly et al. (2010b) [81]
	Beef cattle	118	RFI	Urea	Nascimento et al. (2015) [82]
	Beef cattle	22	RFI	γ-glutamyl transferase, ceruloplasmin, myeloperoxidase, reactive oxygen metabolites, retinol, urea globulin, and zinc	Ferronato et al. (2023) [83]
	Beef cattle	50	RFI	Pantothenate	Clemmons et al. (2019) [84]
	Beef cattle	16	ADG	Alpha-linolenic acids, arachidonic acid, linoleic acid, stearic acid and pentadecanoic acid	Artegoitia et al. (2017) [39]
	Beef cattle	50	RFI	3,4-dihydroxyphenylacetate, 4-pyridoxate, citraconate, hypoxanthine, succinate/methylmalonate, thymine, uracil, and xylose	Clemmons et al. (2020) [85]
	Beef cattle	12	RFI	γ-linolenic acid, 9,10-DHOME, 9-OxoODE, (3S)-3,6-Diaminohexanoate, Glutaric acid, L-proline, L-phenylalanine, L-isoleucine, piperidine, palmitic acid, and S-glutaryldihydrolipoamide	Liu et al. (2022a) [86]
	Beef cattle	52	RFI, FE, and ADGr	Aspartate, C14:0, C22:1n9, choline, dimethylamine, hypoxanthine, and methylamine	Malheiros et al. (2023) [8]
	Beef cattle	25	RFI	Butylcarnitine, carnitine, formate, leucine, lysoPC, propionylcarnitine, serine and SM (20:2)	Foroutan et al. (2020) [36]
	Beef cattle	50	RFI	Carnitine, glutamine, homocysteine and pantothenate	Clemmons et al. (2017) [87]
	Beef cattle	10	RFI	Beta-alanine, choline, L-aspartic acid, L-Glutamate, L-Histidine, L-Isoleucine, L-Lysine, L-Methionine, L-Serine, L-Tyrosine, lysoPC (20:4(8Z,11Z,14Z,17Z)), lysoPC (20:4(5Z,8Z,11Z,14Z)), lysoPC (20:2(11Z,14Z)), lysoPC (18:1(11Z)), lysoPC (18:1(9Z)), lysoPC (16:0), lysoPC (18:0), lysoPC (22:0), PC (18:2(9Z,12Z)/20:4(5Z,8Z,11Z,14Z)), phosphocholine and PS (18:0/20:4(8Z,11Z,14Z,17Z))	Liu et al. (2022b) [88]
	Beef cattle	237	RFI e FCR	Acetylcarnitine, acylethylphosphatidylcholine C36:1, arginine, diacylphosphatidylcholine C32:0, free carnitine, myristylcarnitine, sphingomyelin C20:2, stearoylcarnitine, suberylcarnitine and valerylcarnitine	Widmann et al. (2015) [89]
	Beef cattle	32	ADGr	3-aminopyrazine-2-carboxylate, 8-hydroxyguanosine, adrenochrome o-semiquinone, alanyl-phenylalanine, anhydromarasmone, cyanobutanoic acid, dihydroxy-cholanic acid, hemigossypol, hydroxycinnamic acid, slaframine, and xanthoxylin	Idowu et al. (2023) [90]
	Beef cattle	36	RFI	CO_2_ and urea	Gonano et al. (2014) [91]
	Beef cattle	48	RFI	No significant association	Jorge-Smeding et al. (2022) [92]
	Dairy cattle	473	RFI	NEFA ^1^ and beta-hydroxybutyrate	Marinho and Santos (2022) [93]
	Dairy cattle	29	RFI	Cholecystokinin, NEFA ^1,^ and neuropeptide Y	Xi et al. (2016) [94]
	Dairy cattle	970	RFI	Glucose	Dechow et al. (2017) [95]
	Dairy cattle	188	RFI	Acylcarnitine C3 and acylcarnitine C4	Martin et al. (2021) [38]
	Dairy cattle	10	ECM/DMI	No significant association	Kennedy et al. (2021) [96]
	Dairy cattle	47	RFI	No significant association	Fitzsimons et al. (2014) [97]
	Dairy cattle	36	RFI and ADGr	Beta-hydroxybutyrate	Leao et al. (2021) [98]
	Dairy cattle	18	FCR	2-hydroxyvaleric acid, 2,4-diaminobutyric acid, 4-hydroxybutyrate, 5-aminovaleric acid, lactic acid and lauric acid	Xue et al. (2022) [99]
	Dairy cattle	20	RFI	Alanine, aspartate, fatty acids, and glutamate	Wang and Kadarmideen (2019) [100]
	Dairy cattle	26	RFI	(R)-lipoic acid, 11Z-Eicosenoic acid, 15(S)-HPETE, 15-KETE, 2,4-Dichlorophenol, 2-Arachidonylglycerol, 2-Lysolecithin, 5,10-Methylene-THF, 5-Aminolevulinic acid, 8-Isoprostane, 9,10-DHOME, AICAR, alpha-dimorphecolic acid, androstanedione, arachidonic acid, ascorbic acid, biotin, calcitriol, CE(18:0), CE(22:2(13Z,16Z)), ceramide (d18:1/16:0), ceramide (d18:1/18:0), cervonoyl ethanolamide, chenodeoxyglycocholate, cholesterol ester, chondroitin sulfate, cyclic gmp, cytidine triphosphate, deoxyuridine, diglyceride, D-Urobilin, folinic acid, fumaric acid, galactaric acid, glucuronide, glycocholic acid, guanosine, guanosine triphosphate, hippuric acid, leukotriene E4, L-Tryptophan, L-Urobilin, lysoPC(18:3(6Z,9Z,12Z)), lsoPC(22:1(13Z)), lysoPC(22:2(13Z,16Z)), lysoPC(P-18:1(9Z)), maltotriose, melatonin, muconic acid, nicotinamide ribotide, oleic acid, orotidine, oxitriptan, pregnenolone, prostaglandin F2α, pyridoxal phosphate, sphingosine, S-Sulfo-L-cysteine and UDP-N-acetylglucosamine	Elolimy et al. (2020) [101]
	Dairy cattle	320	RFI	Beta-hydroxybutyrate, bilirubin, ceruloplasmin, glucose, interleukin-6, myeloperoxidase, NEFA ^1^, paraoxonase, and reactive oxygen metabolites	Elolimy et al. (2022) [102]
	Dairy cattle	12	RFI	Butyrate, isovalerate, propionate, total ^4^ SCFA, and valerate	Ben Shabat et al. (2016) [103]
	Dairy cattle	16	RFI	No significant association	Thornhill et al. (2014) [104]
Sheep	Beef ovine	30	RFI	No significant association	Zhang et al. (2017) [105]
	Beef ovine	165	RFI	Acetone, ADMA ^2^, alpha-aminoadipic acid, carnitine, Cesium (Cs), Copper (Cu), glycerol, hexadecanoylcarnitine, hexanoylcarnitine, hippuric acid, hydroxytetradecenoyl carnitine, hydroxyvalerylcarnitine, isopropyl alcohol, ketoleucine, LysoPC at C18:1, LysoPC at C16:1, PC aa C40:2, PC ae C36:0, PC aa C40:1, PC aa C32:2, PC ae C40:6, Potassium (K), spermidine, taurine and valerylcarnitin	Goldansaz et al. (2020) [106]
	Beef ovine	30	RFI	No significant association	Nie et al. (2016) [107]
	Beef ovine	277	RFI	Beta-hydroxyisovalerate, citrate, L-leucine, L-serine, L-threonine, and malate	Touitou et al. (2022) [108]
	Beef ovine	24	FCR	Hydroxylysine and hydroxyisovalerylcarnitine	Kahraman et al. (2022) [109]
	Dual ability	16	FE index	120.0775_2.113, 294.1507_1.669, 327.1317_2.224, 352.2438_9.557, 477.2491_8.272, 521.3352_9.575, 824.4645_8.028, 824.665_8.028, 844.4785_8.052, 1030.8308_8.02, 1031.0809_8.02, 1040.8116_9.53, (3-carboxypropyl)trimethylammonium cation, 5-pyrimidinecarboxamide, 10-hydroxy-9-nitrooctadecanoic acid, alanine, glycine, glutamate, isoleucine, leucine, lysine, L-proline, L-pipecolic acid, (methoxyethoxy-ethoxy)ethyl-aminoundecanoate, nalidixic acid, PA (32:1), PC 20:4e, PE (18:2), PS (o38:2), proline, serine, tributylamine, trans-cinnamic acid (-H2O), trans-cinnamic acid and xanthosine	Toral et al. (2023) [18]
	Dual ability	16	RFI and FEindex	Ammonia, isomers 18:1 and 18:2, lactose, linoleic acid (cis-9,cis-12 18:2), FA trans- 9, cis-12 18:2, FA cis-9 18:1, FA cis-12 18:1, FA trans-6+7+8 18:1, medium-chain unsaturated fatty acid trans-9 16:1, odd-chain fatty acid 17:0, stearic acid, total fatty acids, and very long chain unsaturated fatty acid cis-11 20:1 and 20:2n-6	Toral et al. (2021) [110]
Buffalo	Dairy buffalo	18	RFI	No significant association	Sharma et al. (2016) [75]
Goat	Dairy caprine	30	ADG	(2s)-2-butanol o-[b-d-apiofuranosyl-(1->6)-b-d-glucopyranoside], (r)c(r)s-s-propylcysteine sulfoxide, (r)-s-lactoylglutathionate(1-), 2,3-butanediol glucoside, 2-amino-2-deoxyisochorismate, 2-keto-6-acetamidocaproate, 2-nonenoylglycine, 3,4,5,6-tetrahydrohippuric acid, 3-galactosyllactose, 3-nonenoylglycine, 4-amino-5-hydroxymethyl-2-methylpyrimidine, 7-hydroxyoct-6-enoylglycine, 7-methyl-1,4,5-naphthalenetriol 4-[xylosyl-(1->6)-glucoside], 7-nonenoylglycine, 8-nonenoylglycine, arginyl-proline, asparaginyl-threonine, asp-phe, de-o-methylsimmondsin, ethyl glucuronide, gamma-l-glutamyl-l-pipecolic acid, glucosinalbin, glutamylvaline, isoleucyl-hydroxyproline, isoleucyl-tyrosine, kyotorphin, l-lysopine, l-oxalylalbizziine, l-pyridosine, maltotriose, melibiose, methionyl-serine, n-(7-isocucurbinoyl)isoleucine, n-acetyl-alpha-d-glucosamine 1-phosphate, n-alpha-acetyl-l-arginine, o-succinyl-l-homoserine, phenylalanyl-valine, putrescine, threoninyl-glycine, tyrosyl-isoleucine, valproic acid glucuronide, valyl-hydroxyproline and valyl-isoleucine	Wang et al. (2023) [111]

* N = sample size; ^1^ NEFA: non-esterified fatty acids; ^2^ ADMA: asymmetric dimethylarginine; ^3^ BCFA: branched-chain fatty acids; ^4^ SCFA: short-chain fatty acids.

## Data Availability

The original contributions presented in the study are included in the article/supplementary material; further inquiries can be directed to the corresponding author.

## Supplementary Materials

The following supporting information can be downloaded at: https://www.mdpi.com/article/10.3390/metabo14120675/s1, Table S1: Title, authors, and year of the selected studies in the systematic review.
